# Antipseudomonal and Immunomodulatory Properties of Esc Peptides: Promising Features for Treatment of Chronic Infectious Diseases and Inflammation

**DOI:** 10.3390/ijms22020557

**Published:** 2021-01-08

**Authors:** Floriana Cappiello, Veronica Carnicelli, Bruno Casciaro, Maria Luisa Mangoni

**Affiliations:** 1 Laboratory Affiliated to Pasteur Italia-Fondazione Cenci Bolognetti, Department of Biochemical Sciences, Sapienza University of Rome, P. le Aldo Moro 5, 00185 Rome, Italy; 2 Department of Biotechnological and Applied Clinical Sciences, University of L’Aquila, 67100 L’Aquila, Italy; veronica.carnicelli@univaq.it; 3 Center for Life Nano Science@Sapienza, Istituto Italiano di Tecnologia, Viale Regina Elena 291, 00161 Rome, Italy; bruno.casciaro@iit.it

**Keywords:** antimicrobial peptides, inflammation, interleukin-6, cyclooxygenase-2, wound healing, lipopolysaccharide, *Pseudomonas aeruginosa*

## Abstract

Persistent infections, such as those provoked by the Gram-negative bacterium *Pseudomonas aeruginosa* in the lungs of cystic fibrosis (CF) patients, can induce inflammation with lung tissue damage and progressive alteration of respiratory function. Therefore, compounds having both antimicrobial and immunomodulatory activities are certainly of great advantage in fighting infectious diseases and chronic inflammation. We recently demonstrated the potent antipseudomonal efficacy of the antimicrobial peptide (AMP) Esc(1-21) and its diastereomer Esc(1-21)-1c, namely Esc peptides. Here, we confirmed this antimicrobial activity by reporting on the peptides’ ability to kill *P. aeruginosa* once internalized into alveolar epithelial cells. Furthermore, by means of enzyme-linked immunosorbent assay and Western blot analyses, we investigated the peptides’ ability to detoxify the bacterial lipopolysaccharide (LPS) by studying their effects on the secretion of the pro-inflammatory cytokine IL-6 as well as on the expression of cyclooxygenase-2 from macrophages activated by *P. aeruginosa* LPS. In addition, by a modified scratch assay we showed that both AMPs are able to stimulate the closure of a gap produced in alveolar epithelial cells when cell migration is inhibited by concentrations of *Pseudomonas* LPS that mimic lung infection conditions, suggesting a peptide-induced airway wound repair. Overall, these results have highlighted the two Esc peptides as valuable candidates for the development of new multifunctional therapeutics for treatment of chronic infectious disease and inflammation, as found in CF patients.

## 1. Introduction

It is largely documented that the Gram-negative bacterium *Pseudomonas aeruginosa* is the principal etiological agent of chronic pulmonary infections in cystic fibrosis (CF) patients, where it colonizes the lung environment and infects cells, which leads to deterioration of lung tissue and failure of respiratory function [1,2,3]. Furthermore, it is known that the lipopolysaccharide (LPS or endotoxin), the major constituent of the outer membrane of Gram-negative bacteria, is released from the bacterial cell wall following bacterial cells division or death and upon antibiotic treatment. The LPS is recognized as a pathogen-associated molecular pattern by specific receptors expressed on immune cells, i.e., Toll-like receptors (TLRs), which are then activated. In particular, the LPS interacts with monocytes and macrophages through TLR4 and plays a crucial role in the inflammation process by inducing the production of pro-inflammatory cytokines, such as tumor necrosis factor-α (TNF-α), interleukin (IL)-6, and the expression of the enzyme cyclooxygenase-2 (COX-2) which controls the formation of several inflammatory mediators catalyzing conversion of arachidonic acid to different prostaglandins including prostaglandin E2 [4,5,6,7,8]. Notably, inflammation is the primary physiological response of the immune system to external and internal stimuli (e.g., infections or tissue injuries), a response that restores the integrity of damaged tissues; the regulation of inflammation is essential for the tissue homeostasis in damaged biological compartments [9,10,11,12].

However, an excessive and dysregulated production of inflammatory molecules (which occurs, for example, during persistent infections maintained by TLRs signaling) can become harmful and can lead to septic shock syndrome in most serious cases, increasing the risk of mortality for affected people [11,13,14]. Therefore, compounds having both antimicrobial and immunomodulatory activities are certainly of great advantage to fight infectious diseases and chronic inflammation.

In this regard, antimicrobial peptides (AMPs) hold promise. They are a diverse group of evolutionarily conserved small bioactive molecules in most living species, and they display a critical function in the host innate immunity, acting as part of the first defense line against invading microorganisms [2,15].

In comparison with conventional antibiotics, they have a quick and broad spectrum of activities against various Gram-negative and Gram-positive bacteria, viruses, fungi, and parasites, with low tendency to induce resistance. In addition, they are endowed with immunoregulatory properties encompassing immune cell differentiation, LPS neutralization, promotion of angiogenesis, and stimulation of tissue repair [9,16,17], through multiple mechanisms including the modulation of TLRs-mediated pathways [2,18,19,20,21,22].

Recently, we identified two N-terminal derivatives of the frog skin AMP esculentin-1a, esculentin-1a(1-21)NH_2_ [Esc(1-21) GIFSKLAGKKIKNLLISGLKG-NH_2_] and its diastereomer Esc(1-21)-1c carrying d-Leu14 and d-Ser17 (Esc peptides), with potent in vitro and in vivo activity against both the free living and biofilm form of *P. aeruginosa* [23,24,25]. Indeed, Esc peptides gave rise to the following: (i) ≥3 log_10_ reduction of the number of viable planktonic bacterial cells compared to untreated samples, at a concentration of 1 μM and 4 μM for the all-L peptide and the diastereomer, respectively; (ii) 95% reduction in the number of viable biofilm cells at a concentration range between 12 and 25 μM; (iii) ∼3 × 10^4^ or ∼4.7 × 10^3^ reduction of lung bacterial burden in a mouse model of acute *P. aeruginosa* lung infection when 0.1 mg/kg of Esc(1-21) or Esc(1-21)-1c were intratracheally administered [23,24,25].

In addition, both peptides were found to inhibit the release of TNF-α from macrophages activated by the *P. aeruginosa* LPS, presumably through their physical interaction with the endotoxin. This event would result in the breakage of LPS aggregates, the biologically active form of the LPS, into smaller particles [26,27,28], preventing LPS-TLR4-mediated activation of immune cells which is expected to avoid an exaggerated inflammatory response [23]. Furthermore, it was discovered that Esc(1-21) and its diastereomer can promote migration of lung epithelial cells [23] also in the presence of the *P. aeruginosa* LPS to mimic a bacterial infection condition [29]. This should favor the recovery of entirety and functionality of lung tissue that is highly compromised following persistent infections.

Here, besides exploring the ability of Esc peptides to kill *P. aeruginosa* once internalized into alveolar basal epithelial cells (i.e., A549 cells), we expanded our knowledge on their immunomodulatory features by investigating their effects (i) on the secretion of the pro-inflammatory cytokine IL-6 and the expression of COX-2 from macrophages activated by the *P. aeruginosa* LPS as well as (ii) on the stimulation of airway wound repair under conditions simulating infections by monitoring the closure of a gap produced in A549 cells upon exposure to the *P. aeruginosa* LPS. Overall, our results have contributed to the making of these peptides as new valuable alternative compounds for the development of anti-infective and immunomodulatory therapeutics, especially for treatment of chronic infectious diseases, as in CF sufferers.

## 2. Results

### 2.1. Effect of Esc Peptides on A549 Cells Infected with Pseudomonas aeruginosa ATCC 27853

**Figure 1:**
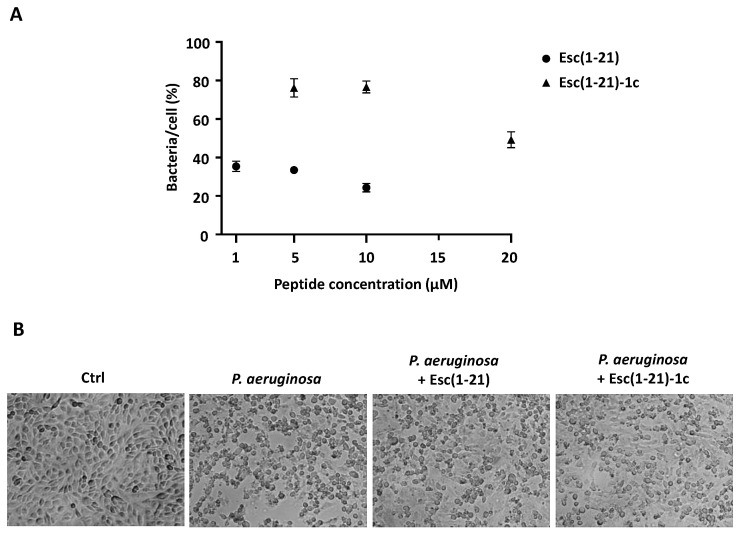
Effect of Esc(1-21) and its diastereomer Esc(1-21)-1c on *Pseudomonas aeruginosa* ATCC 27853 infected A549 cells. About 1 × 10^5^ cells were seeded in 24-well plates. Upon reaching confluence, they were infected with *P. aeruginosa* for 2 h and then antibiotic treatment was performed to remove nonadherent extracellular bacteria. Afterwards, infected cells were left untreated or were treated for 1 h with each peptide at different concentrations. (**A**) The percentage of bacteria/cell was calculated with respect to untreated infected samples. All data are the mean from four independent experiments ± standard errors of the means (SEM). (**B**) Light microscopy images of A549 cells at ×10 magnification. Peptide-untreated uninfected cells are control samples (Ctrl).

It is well known that binding of *P. aeruginosa* to airway epithelial cells, mediated by bacterial products such as LPS, flagella, pili, alginate, and by some host cell receptors including asialoGM1, TLRs, fibronectin, and α5β1 integrin, can result in bacteria internalization [30,31,32]. In this regard, we evaluated the antibacterial activity of Esc(1-21) and its diastereomer on A549 cells infected with the reference strain of *P. aeruginosa* ATCC 27853 by counting the number of surviving bacteria, upon peptide treatment. The number of intracellular bacteria, expressed as percentage of colony forming units (CFU) for each infected cell, was calculated with respect to peptide untreated samples. Both peptides significantly reduced the percentage of internalized bacteria per cell (Figure 1A). Esc(1-21) caused about a 65% reduction of CFU at 1 and 5 µM; 75% at 10 µM, while the diastereomer provoked about 25% reduction of viable bacterial cells at 5 and 10 µM; 50% at 20 µM (Figure 1A). As pointed out by optimal microscopy analysis (Figure 1B), disruption of A549 monolayer induced by *P. aeruginosa* infection appeared to be partially prevented when epithelial cells were treated with each Esc peptide.

### 2.2. Effect of Esc Peptides on IL-6 Secretion and COX-2 Expression in Macrophages Stimulated by Pseudomonas aeruginosa LPS

**Figure 2:**
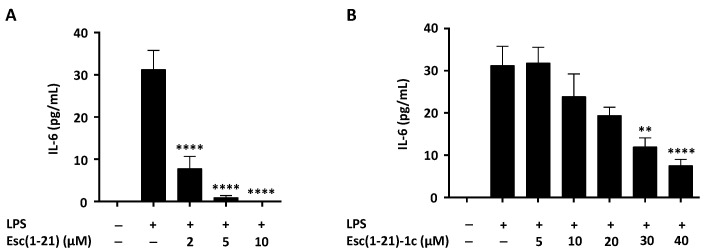
Effect of Esc(1-21) (**A**) or Esc(1-21)-1c (**B**) on IL-6 release from LPS-activated RAW 264.7 macrophages. Cells were stimulated for 4 h with *P. aeruginosa* LPS (20 ng/mL) alone or in combination with peptides at the indicated concentrations. The level of IL-6 in supernatants was measured by using enzyme-linked immunosorbent assay. Values are expressed as the mean ± SEM of three independent experiments. The level of statistical significance between samples treated with the LPS and those stimulated with the LPS plus a peptide was determined by one-way ANOVA analysis and is indicated as follows: ** *p* < 0.01 and **** *p* < 0.0001.

To investigate the immunomodulatory properties of Esc peptides, we initially analyzed their LPS-neutralizing activity by monitoring the peptides’ capability to inhibit or not inhibit the release of IL-6 from macrophages. These cells were activated by the *P. aeruginosa* LPS for 4 h in the absence or presence of various concentrations of Esc(1-21) or Esc(1-21)-1c, as indicated in Figure 2A,B. While the LPS induced a significant IL-6 secretion with respect to untreated cells, both peptides, when combined with the LPS, significantly reduced IL-6 extracellular release in a dose-dependent manner with a more pronounced effect for Esc(1-21). Interestingly, this latter component completely abolished the secretion of this cytokine at a concentration of 10 µM. In parallel, untreated control cells (Figure 2) and cells treated with the peptides alone (data not shown) did not induce any detectable IL-6 secretion.

**Figure 3:**
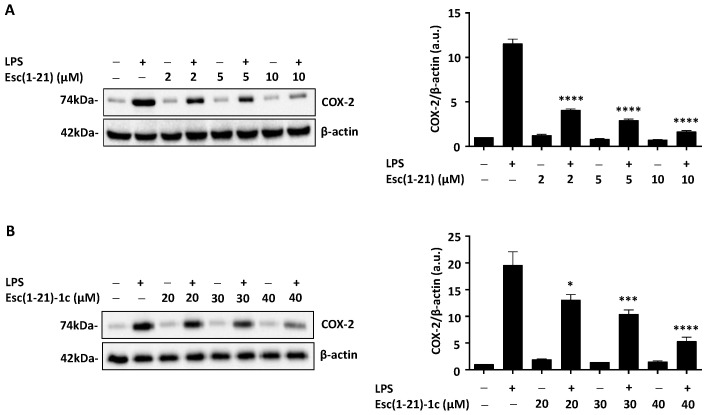
Effect of Esc(1-21) (**A**) or Esc(1-21)-1c (**B**) on COX-2 expression in LPS-stimulated RAW 264.7 macrophages. Cells were treated with *P. aeruginosa* LPS (20 ng/mL), peptides or LPS in combination with peptides as indicated, for 4 h. β-actin was used as loading control (cropped images). Samples we ran in the gels derive from the same experiment and were processed in parallel. Molecular weights of COX-2 and β-actin are also indicated. Full-length images are reported in Supplementary Figure S1. The right panels show quantitative analysis of COX-2/β-actin ratio as measured by densitometry scanning using ImageJ software. Data are expressed in arbitrary units (a.u.) and are the mean ± SEM of three independent experiments. The level of statistical significance between samples treated with the LPS and those stimulated with the LPS plus peptide was determined by one-way ANOVA analysis and is indicated as follows: * *p* < 0.05; *** *p* < 0.001 and **** *p* < 0.0001.

Activation of the immune system is triggered not only by interleukins but also by intracellular compounds including COX-2 [18,33,34]. Because several AMPs have been reported to reduce the expression of this enzyme [7,35], we decided to evaluate the amount of COX-2 in macrophages exposed to the LPS from *P. aeruginosa*, with or without Esc(1-21) or Esc(1-21)-1c at different concentrations. The results of Western blot analysis (Figure 3) highlighted that treatment of macrophages with the LPS up-regulated COX-2 protein expression, whereas Esc(1-21) (Figure 3A) and its diastereomer (Figure 3B) led to a consistent lowering of the expression level of COX-2, in a dose-dependent manner. As for IL-6, a marked effect was detected for Esc(1-21) which approximately induced a three-fold reduction of COX-2 levels when used at a concentration of 2 µM in combination with the LPS, while a similar reduction was observed for Esc(1-21)-1c at a dose of 40 µM. No appreciable alteration of COX-2 expression was observed between untreated control samples and samples treated with the peptides alone (Figure 3).

### 2.3. Wound Healing Assay and Dimensional Analysis in the Presence of the LPS or Its Combination with Peptides

**Figure 4:**
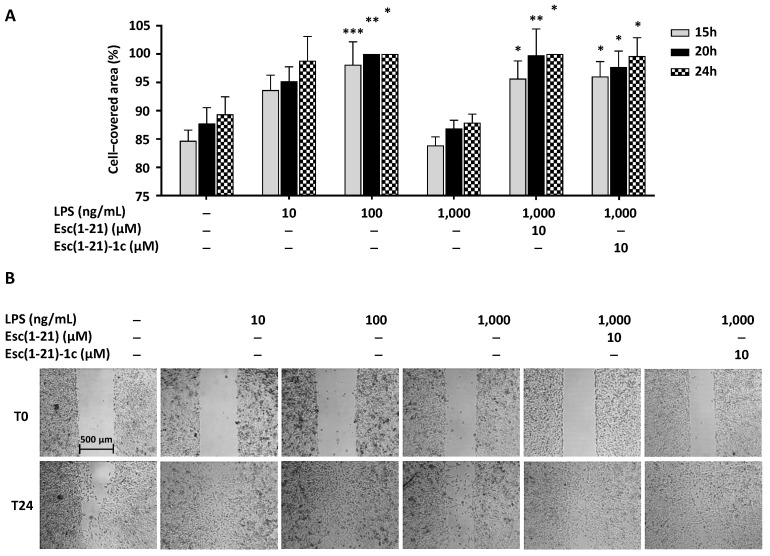
(**A**) Effect of LPS alone or LPS in combination with Esc(1-21) and its diastereomer Esc(1-21)-1c on the closure of a pseudowound field produced in a monolayer of A549 cells. A549 cells were seeded in each side of an Ibidi culture insert. After reaching confluence, they were treated or not with the LPS or the LPS plus peptide, as indicated. Cells were photographed at the time of insert removal (T0) and checked for cell migration after 15, 20, and 24 h. The percentage of the cell-covered area at each time point is indicated on the *y*-axis. Control (Ctrl) represents cells not treated with the peptide or the LPS. All data are the mean of three independent experiments ± SEM. The level of statistical significance between Ctrl and LPS-treated samples or between samples treated with 1000 ng/mL LPS and LPS plus peptide was determined by two-way ANOVA analysis and is indicated as follows: * *p* < 0.05; ** *p* < 0.01 and *** *p* < 0.001. (**B**) The micrographs show representative results of pseudowound closure induced after 24 h of the LPS treatment with respect to that of Ctrl sample or the LPS in the presence of peptides with respect to that of LPS-treated samples.

Analogous to what was previously obtained with bronchial cells [29], the LPS promoted the closure of a gap produced in a monolayer of A549 cells already within 15 h at an optimal concentration of 100 ng/mL (Figure 4A). Differently, when the amount of the LPS was increased to 1000 ng/mL, cell migration was basically hampered (Figure 4A). We can exclude that this outcome is due to the cytotoxicity of the LPS, as the percentage of metabolically active cells detected in a viability assay after treatment with the LPS at a concentration of up to 1000 ng/mL was similar to that of untreated control samples (data not shown).

However, when each Esc peptide, at 10 µM, was incubated with the LPS at the concentration that curbed the wounded field closure (1000 ng/mL), cell migration was clearly restored during 15 h of treatment (Figure 4A). Figure 4B shows representative micrographs of A549 pseudowound before (time 0, T0) and 24 h after incubation with the LPS in the presence of the peptides or not. In addition, at this time interval the difference between the cell-covered area of peptide-treated samples versus that of samples treated with the LPS alone was statistically significant.

**Figure 5:**
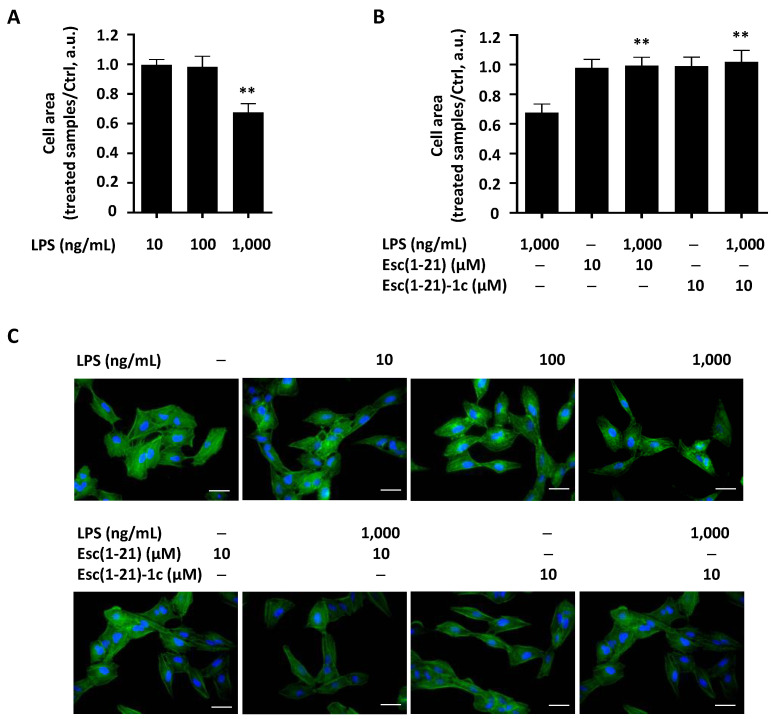
Dimensional analysis of A549 cells after 24 h treatment with the LPS at different concentrations (**A**) or following stimulation with Esc peptides alone or in combination with the LPS at 1000 ng/mL (**B**). Cells were stained with phalloidin and analyzed by ImageJ program. The results are reported as the ratio between the area of treated and untreated Ctrl samples ± SEM. The level of statistical significance between Ctrl and LPS-treated samples or between the LPS in combination with peptides and the LPS alone was determined by one-way ANOVA analysis and is indicated as follows: ** *p* < 0.01. (**C**) Representative micrographs showing the effects of the LPS, peptides, or their combination on A549 cells dimensions. Bars are 20 μm long.

Moreover, we carried out morphological studies by staining A549 cells with phalloidin. Using fluorescence microscopy analysis, we observed that incubation of cells with 1000 ng/mL LPS was accompanied by a reduction in cell dimensions with respect to control samples or those incubated with the LPS at a lower concentration (i.e., 10 ng/mL or 100 ng/mL) (Figure 5A). In comparison, according to the wound healing results, when cells were treated with 1000 ng/mL of the LPS in the presence of each peptide (at a concentration of 10 µM), cell dimension reverted to that of control samples (Figure 5B). Representative images of the effects of the LPS, peptides, or their combination on cell morphology after 24 h of treatment are shown in Figure 5C.

## 3. Discussion

The inflammatory response to airborne microbial pathogens involves both immune cells like macrophages and airway cells like alveolar and bronchial epithelial cells [36,37]. During Gram-negative bacteria infections and after conventional antibiotic exposure, the spontaneous and rapid release of the LPS elicits the activation of the host innate immune system.

The TLRs interaction with microbial components such as the LPS triggers the intracellular signaling cascade which ends with the translocation of the transcription factor NF-κB into the nucleus followed by the upregulation of COX-2 enzyme and the release of pro-inflammatory cytokines to eliminate the source of inflammatory stimuli and to renew homeostasis [21,37,38]. However, TLRs-dependent biochemical pathways have to be finely controlled to prevent excessive inflammation and cell necrosis. As an example, persistent *P. aeruginosa*-induced pulmonary infection in CF patients leads to chronic airway inflammation with structural lung tissue damage [39,40,41]. Several reports have described elevated concentrations of pro-inflammatory cytokines, e.g., IL-6, in the sputum and bronchoalveolar lavage fluid of CF sufferers [42,43], and these high levels reflect the state of disease of CF airways [44]. In this scenario, thanks to their broad-spectrum of antimicrobial activities and immunomodulatory properties [45], AMPs represent attractive compounds for the development of new therapeutics with dual antibacterial and anti-inflammatory functions.

We previously reported on the in vitro and in vivo antipseudomonal efficacy of Esc(1-21) and Esc(1-21)-1c AMPs [23,24,25]. Note that *P. aeruginosa* strains can escape the host immune system by invading airway epithelial cells and surviving inside these cells [30]. Here, we demonstrated the killing of *Pseudomonas aeruginosa* internalized into A549 cells upon 1 h of exposure to Esc peptides. Moreover, the integrity of *P. aeruginosa*-infected cells’ monolayers was partially maintained upon addition of the peptides. In our previous work, by means of rhodamine-labeled peptides and confocal microscopy analysis, we showed that both Esc peptides are able to enter bronchial epithelial cells [29]. Although the mechanism underlying the killing activity of intracellular bacteria by both AMPs is not yet known, we can hypothesize that this event is the result of a direct interaction of the internalized peptides with the intracellular bacterial cells. However, activation of other cellular processes controlling host cell protection from microbial pathogens may be involved.

In this study, we have also discovered that Esc peptides highly reduce IL-6 production and COX-2 expression in LPS-stimulated macrophages. The IL-6 inhibition supports the effect of Esc peptides on the impaired secretion of the other pro-inflammatory cytokine (i.e., TNF-α) from the same cells [23]. Macrophages are protagonist immune cells because they move towards the site of infection either to neutralize the pathogens or to restore cell physiology. The COX-2 enzyme catalyzes conversion of arachidonic acid to prostaglandin E2. This latter is a fundamental inflammatory mediator whose synthesis must be modulated in order to limit an overstate production [46]. Therefore, the Esc-peptides-induced downregulation of both IL-6 and COX-2 in LPS-stimulated macrophages represents a favorable feature to confer the peptides the ability to impede the development of chronic inflammation.

One of the mechanisms underlying this finding likely relies on the disaggregation of LPS molecules by the Esc peptides [23]. However, other events can imply the inhibition of TLR-4 endocytosis in LPS-stimulated macrophages [47] and the attenuation of nuclear translocation of NF-kB, as already described for other peptides [18]. Remarkably, in addition to their function as pathogen recognition receptors, TLRs play a key role in promoting tissues structural repair. In fact, inflammation and wound healing processes are closely related [48]. The expression of TLR4 on alveolar epithelial cells type II, e.g., A549 cells, that are in charge for alveolar damage repair during infection and tissue injury [49] is a fundamental hallmark for cell regeneration (self-renewal) and wound repair mechanisms [32,50]. As already proven in bronchial epithelial cell lines [29], we confirmed that high concentrations of the LPS mimicking an infectious condition impair the re-epithelialization of the airway epithelium, using A549 cells as experimental model. However, treatment of these cells with Esc peptides (at a concentration of 10 µM) allowed repristinating of cell migration and presumably to heal airway wounds. The inhibitory effect of high LPS concentrations on cell migration is likely related to its influence on cell cytoskeleton. In fact, as reported in the literature, the LPS has a role in the cytoskeleton remodeling and each cell type is differently affected. Contrary to what occurs in macrophages and monocytes, the LPS induces depolymerization of microfilaments by reorganization of F-actin in alveolar epithelial cells [51,52,53]. In particular, following LPS stimulation, the periphery-located F-actin is replaced by centralized stress fibers, lamellipodial structures are reduced or lost, while focal adhesions appear and the regularly shaped cells connected to each other become irregular [32,54]. By morphological analyses we pointed out that Esc peptides have the capability to recondition cellular dimensions that are altered in the presence of high LPS concentrations.

## 4. Materials and Methods

### 4.1. Peptides Synthesis

Synthetic Esc(1-21) and its diastereomer, Esc(1-21)-1c, were purchased from Biomatik (Wilmington, NC, USA). Stepwise solid-phase synthesis using a standard F-moc strategy was carried out to assemble each peptide subsequently purified via reverse-phase high-performance liquid chromatography (RP-HPLC) to a purity of 98%. The molecular mass was checked by mass spectrometry.

### 4.2. Cell Culture and Microorganisms

The murine RAW 264.7 macrophage cell line and the human type II alveolar epithelial cell line A549 were obtained from the American Type Culture Collection (ATCC, Manassas, VA, USA) [55]. The culture medium employed for both cell lines was Dulbecco’s modified Eagle’s medium (DMEM) containing 2 mM glutamine (DMEMg) and supplemented with 10% heat-inactivated fetal bovine serum (FBS), and antibiotics (0.1 mg/mL of penicillin and streptomycin). Non-essential amino acids (NEAA) and sodium pyruvate (1 mM) were added for macrophages. Cell culture materials were purchased from Euroclone (MI, Italy). Cell lines were grown in 25-cm^2^ and 75-cm^2^ flasks at 37 °C in a humidified atmosphere with 5% CO_2_. For cell infection, *P. aeruginosa* ATCC 27853 strain [56] was employed.

### 4.3. Cell Infection and Peptide Effect on Intracellular Bacteria

About 1 × 10^5^ A549 cells resuspended in DMEMg supplemented with 10% FBS were seeded in each well of 24-well plates and grown to confluence for 2 days at 37 °C and 5% CO_2_. *P. aeruginosa* ATCC 27853 was grown in Luria-Bertani broth at 37 °C with mild shaking (125 rpm) to mid-log phase (optical density of 0.8 at λ = 590 nm) and subsequently centrifuged. The pellet was resuspended in DMEMg and then syringed using a 21-gauge needle to avoid bacteria aggregation. To infect A549 cells a multiplicity of infection (MOI) of 100:1 (bacteria to cells) was employed. More precisely, two hundred microliters of bacterial suspension (containing about 1 × 10^7^ CFU) was incubated for 2 h with A549 cells at 37 °C and 5% CO_2_. After infection, the medium was removed, the cells were washed three times with DMEMg and then treated for 1 h with 200 µg/mL gentamicin in DMEMg plus 2% FBS to remove extracellular bacteria. Afterwards, the medium was aspirated and the infected cells were washed three times as described above. Two hundred microliters of Hanks’ solution (136 mM NaCl, 4.2 mM Na_2_HPO_4_, 4.4 mM KH_2_PO_4_, 5.4 mM KCl, 4.1 mM NaHCO_3_, pH 7.2, supplemented with 20 mM D-glucose) with or without the peptide at different concentrations was added to each well, and the plate was incubated for 1 h at 37 °C and 5% CO_2_. After peptide treatment, each well was washed with phosphate-buffered saline (PBS); afterwards, 300 µL of 0.1% Triton X-100 (Sigma-Aldrich, St. Luis, MO) in PBS were added to each well for 15 min at 37 °C and 5% CO_2_ to lyse A549 cells. Each sample was then sonicated in a water bath for 5 min to break up possible bacterial clumps, and appropriate aliquots were plated on agar plates for CFU counting after 24 h at 37 °C. In parallel, the number of A549 was evaluated before and after bacterial infection and peptide treatment. Briefly, cells were detached from each well by adding 20 μL of TrypLE Express (Gibco, MI, Italy). After 15 min of incubation at 37 °C and 5% CO_2_ an appropriate aliquot was counted under an optical microscope.

### 4.4. Detection of IL-6 Release from RAW 264.7 Macrophages

About 1 × 10^5^ RAW 264.7 cells, suspended in DMEMg supplemented with NEAA, sodium pyruvate and 10% FBS, were seeded in each well of 96-well plates. After overnight incubation at 37 °C and 5% CO_2_, the medium was removed and replaced with fresh medium plus 10% FBS containing 20 ng/mL LPS derived from *P. aeruginosa* (Sigma-Aldrich, St. Luis, MO) or each Esc peptide at different concentration or LPS in combination with each peptide. Samples were incubated at 37 °C and 5% CO_2_ for 4 h. At the end of the treatment, the supernatants were collected and IL-6 concentration was evaluated by enzyme-linked immunosorbent assay (ELISA), according to the manufacturer’s protocol (Mouse IL-6 ELISA MAX Deluxe set, Biolegend, San Diego, CA, USA). Cells stimulated with LPS alone and untreated cells served as controls.

### 4.5. Western Blotting Analysis

RAW 264.7 macrophages suspended in DMEMg supplemented with NEAA, sodium pyruvate and 10% FBS were seeded in 90 mm plates (1.5 × 10^6^ cells/plate) and cultured overnight at 37 °C and 5% CO_2_. The medium was then removed and cells were treated with LPS from *P. aeruginosa* (20 ng/mL), or each Esc peptide at different concentrations or LPS in combination with each peptide for 4 h. Afterwards, the cells were washed and solubilized in lysis buffer [PBS 1X, 1% Tergitol-type NP-40, 0.5% sodium deoxycholate, 0.1% sodium dodecylsulfate (SDS), Protease Inhibitor Cocktail, 1 mM sodium orthovanadate, 5 mM Dithiothreitol] for 30 min on ice. The samples were centrifuged at 1000× *g* for 10 min and the supernatants were collected. Twenty μg of lysate protein from each sample were separated by SDS-PAGE on 10% gels and transferred to polyvinylidene fluoride membrane. After overnight blocking with buffer (5% non fat dry milk) the membranes were incubated with primary anti-COX-2 or anti-β-actin antibody for 1 h, washed in tris buffered saline-Tween 20 and incubated with horseradish peroxidase-conjugated secondary antibody for 1 h. Blots were developed by enhanced chemiluminescent system. Densitometry analyses of bands were done by ImageJ gel system.

### 4.6. Pseudowound Healing Assay

To evaluate the effect of *P. aeruginosa* LPS alone or in combination with Esc peptides on A549 cells migration, the following protocol was carried out as an alternative to the traditional scratch method [57]. Silicone Ibidi culture inserts were placed in each well of a 12-well plate and A549 cells, suspended in DMEMg supplemented with 10% FBS, were seeded in each side of the inserts at a density of 4 × 10^4^ cells/side. Then, the plate was incubated at 37 °C and 5% CO_2_ to allow cells growing to confluence. After approximately 24 h, the inserts were removed, a cell-free area (pseudowound) of approximately 500 µm was obtained in each cell monolayer, and DMEMg supplemented with 2% FBS with or without LPS, peptides or their combination, at different concentrations, were added to each well. Plate was incubated as reported above and cell migration was visualized at different time intervals under an inverted microscope (Olympus CKX41) at ×4 magnification and photographed with a Color View II digital camera. WIMASIS Image Analysis program was employed to calculate the percentage of cell-covered area.

### 4.7. Fluorescence Microscopy

A549 cells (1.5 × 10^5^), suspended in DMEMg supplemented with 10% FBS, were seeded into 35-mm dish plates where 0.13- to 0.17-mm-thick coverslips were previously placed. Plates were incubated overnight at 37 °C and 5% CO_2_; subsequently, A549 were treated with LPS from *P. aeruginosa* at different concentrations, or with 10 µM Esc peptide or LPS in combination with peptides in DMEMg supplemented with 2% FBS, as indicated. After 24 h incubation, cells were washed with PBS, fixed with 4% formaldehyde for 10 min at 4 °C, washed with PBS and permeabilized with 0.1% Triton X-100 in PBS for 10 min at room temperature. Afterwards, cells were washed again and stained with phalloidin-fluorescein isothiocyanate (40 μM in PBS) for 30 min at room temperature to visualize the cytoskeleton. The nuclei were stained with DAPI (1 µg/mL), which was added to the cells for 5 min at room temperature. The coverslips were mounted on slides using Mowiol mounting medium, observed under the fluorescent microscope KOZO OPTICS XJF800 at ×20 magnification and photographed with a Color View II digital camera. For each sample we selected 30 cells from pictures acquired in three different experiments (about 10 cells/picture). We calculated area of single cells using ImageJ software and we reported the results as ratio between the area of treated and untreated Ctrl samples.

### 4.8. Statistical Analysis

Quantitative data derived from independent experiments were expressed as the mean ± SEM. Statistical significance was determined by one-way or two-way ANOVA analysis with Bonferroni test, as indicated, using PRISM software (GraphPad, San Diego, CA, USA). *p* values of <0.05 were considered statistically significant. The levels of statistical significance are indicated in the legend to the figures.

## 5. Conclusions

All together these data have contributed to emphasizing the potential of Esc peptides as novel drugs not only able to eliminate bacterial pathogens but also able to recover the integrity of a damaged tissue and to display anti-inflammatory activity. These advantageous properties should warrant a successful treatment of chronic infectious diseases and chronic inflammation as found in people affected by CF. Furthermore, recent studies have described a protective role of AMPs produced by nonimmune cells in preventing the development of autoimmune diseases [58]. Therefore, AMPs like Esc peptides represent attractive compounds for the generation of new therapeutics able to modulate and/or arrest inflammation in autoimmunity [58], thus limiting the progression of immune-related diseases [58,59,60].

## Data Availability

Data sharing is not applicable.

## Supplementary Materials

Supplementary Materials can be found at https://www.mdpi.com/1422-0067/22/2/557/s1.

## Abbreviations

AMPs	Antimicrobial peptides
CFU	Colony forming unit
COX-2	Cyclooxygenase-2
DMEM	Dulbecco’s modified Eagle’s medium
DMEMg	Dulbecco’s modified Eagle’s medium supplemented with 2 mM glutamine
ELISA	Enzyme-linked immunosorbent assay
FBS	Fetal bovine serum
IL-6	Interleukin 6
LPS	Lipopolysaccharide
NEAA	Non-essential amino acids
PBS	Phosphate-buffered saline
RP-HPLC	Reverse-phase high-performance liquid chromatography
TLRs	Toll-like receptors
TNF-α	Tumor necrosis factor-α
