# Supplementary material for: Antipseudomonal and Immunomodulatory Properties of Esc Peptides: Promising Features for Treatment of Chronic Infectious Diseases and Inflammation
Source: Int J Mol Sci. 2021 Jan 8;22(2):557. doi: 10.3390/ijms22020557 (PMC7826692; doi:10.3390/ijms22020557)
Supplement: Supplementary file 1 [file ijms-22-00557-s001.pdf]

Supplementary data

Antipseudomonal and immunomodulatory properties of Esc peptides: promising features for treatment of chronic infectious diseases and inflammation

Floriana Cappiello [1,*], Veronica Carnicelli [2], Bruno Casciaro [3] and Maria Luisa Mangoni [1,*]

[1] Laboratory affiliated to Pasteur Italia-Fondazione Cenci Bolognetti, Department of Biochemical Sciences, Sapienza University of Rome, P.le Aldo Moro 5, 00185 Rome, Italy (F.C., M.L.M.);

[2] Department of Biotechnological and Applied Clinical Sciences, University of L'Aquila, 67100 L'Aquila, Italy (V.C.);

[3] Center For Life Nano Science@Sapienza, Istituto Italiano di Tecnologia, Viale Regina Elena 291, 00161 Rome, Italy (B.C.)

* Correspondence: floriana.cappiello@uniroma1.it (F.C.); marialuisa.mangoni@uniroma1.it (M.L.M.); Tel.: +39 0649910838

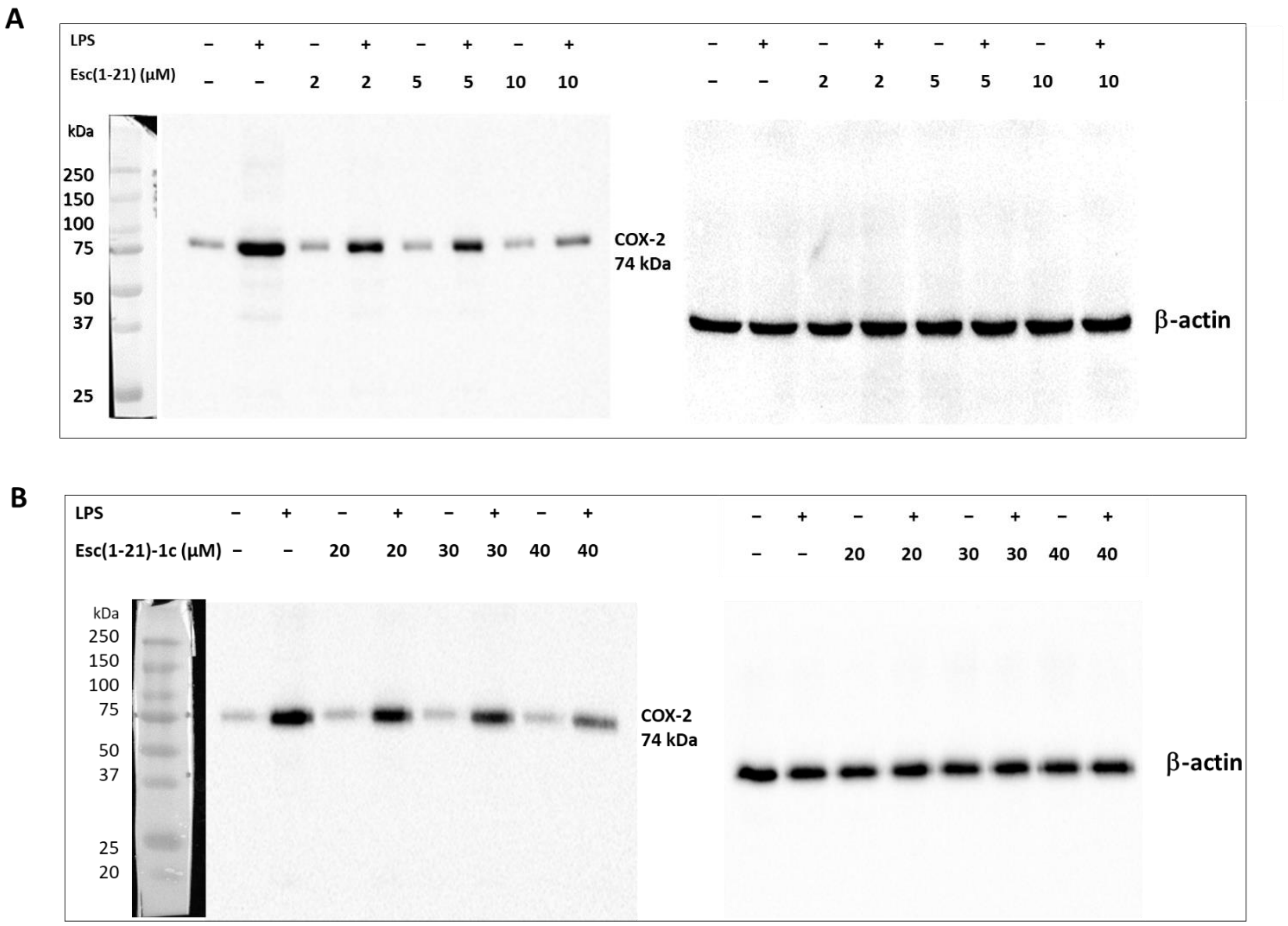

Figure S1.

Western blots show the expression of COX-2 and β-actin proteins in RAW 264.7 macrophages stimulated with *P. aeruginosa* LPS (20 ng/mL), Esc peptides or LPS in combination with peptides as indicated, for 4 h.

Panels A and B show the results obtained when Esc(1-21) and Esc(1-21)-1c were used, respectively. Molecular weights were detected with Amido Black staining solution. Samples showed in the same panel derive from the same experiment and were processed in parallel.
